# Cases Illustrative of the Successful Employment of Oil of Turpentine, in Puerperal Tympanitis, &c.

**Published:** 1853-03

**Authors:** Joseph Smith

**Affiliations:** Danville, Kentucky


*Cases Illustrative of the successful employment of Oil of Turpentine, in Puerperal Tympanitis, &c.* By JOSEPH SMITH, M. D., of Danville, Kentucky.

I submit the following cases to the profession, in which the oil of turpentine was advantageously employed in various puerperal affections, accompanied by tympanitis, and symptoms of incipient metritis, or peritonitis. I do not advance that the cases in question were the typhoid puerperal fever; and my object is simply to call attention to what I deem the undoubted value of the remedy in question, in various forms of abdominal disturbance after labor.

1. Mrs. H. was confined July 10th, 1850. The birth was premature, but there was nothing unusual in the labor. She seemed to be doing well, until the next day; when, on calling, I found her abdomen much enlarged, and very tender, and painful. The lochial discharge was not suppressed; the pulse not over 85; and the skin not hot. Believing the case one of simple tympanitis, I directed the oil of turpentine and castor oil, of each a teaspoonfull every four hours, with fomentations to the abdomen.

After some three or four doses had been taken, the swelling and pain were almost entirely dissipated. There were some partial relapses on the 13th and 14th, which were readily relieved by a dose or two of the same mixture. This combination caused one or two actions from the bowels, more laxative than purgative.

2. Mrs. S. was confined May 21st, 1852, with a natural labor. On the third morning, the 23d, she imprudently rose in bed, and exerted herself. She was taken immediately with a most violent chill, which lasted some two or three hours. Some three hours after the attack I saw her, and found her with hot skin, pulse 120, thirsty, womb very hard, and exceedingly tender and painful. Abdomen not swelled, excepting in the right groin, which was also very tender to the touch. Great restlessness and anxiety of countenance. Lochia and milk both present. This patient I knew from constitutional peculiarities could not use mercurials or opiates; and, as I thought her too weak to bear the lancet, I concluded to try the oil of turpentine. She was directed to take thirty drops of this medicine every two hours, with hot fomentations to the bowels. It was given in a spoonfull of buttermilk.

Being with the case pretty constantly, I was enabled to watch its course. From the second dose, there was evidently some relief of pain and heat, in eight hours there was a decided change, in twenty-four hours there was scarcely a vestige of pain or fever. From that time the turpentine was gradually withdrawn. No relapse.

3. A negro woman of Mr. S.'s was confined about the 25th of July last. Did not attend her. She was reported as doing very well, until August 3d, when I was called to see her. I found her complaining much of her abdomen; the womb was enlarged, hard and tender; pulse 95; tongue coated; skin dry and warm. She had been complaining several days; said the lochia had ceased the night before; some milk. Gave calomel and ipecac. pretty freely, and directed fomentations.

*August 4th.* Found her in much the same condition as on yesterday, only the abdomen more enlarged and painful; stomach still irritable; no milk. Directed fomentations, spirits turpentine 30 drops, with a teaspoonful of paregoric every two hours. This was continued until the 6th, when the stomach became so irritable that the turpentine was suspended. But there was evident and great relief of pain, swelling and fever. Mustard, &c., soon relieved the stomach, and the patient gradually improved until the 12th and 13th; she had a slight relapse, when the turpentine and paregoric was again given, with early relief. This patient took one or two mercurial potions during her attack.

4. Mrs. S—— was confined, Sept. 15th, 1852. First child, easy labor. On the 22d was taken with a severe chill, followed by much pain in abdomen; the womb hard and painful, and strong bearing down sensations, almost like strong labor pains. Of this there has been a good deal since her confinement. Her skin hot and moist; pulse ranging from 120 to 130; thirsty; very restless. Directed spirits turpentine, 30 drops, laudanum, 20 drops, every three hours; fomentations. In twenty-four hours the fever, pain and swelling were almost entirely gone. The bearing down tenesmus in this case was very peculiar. It was present at intervals for ten days after labor, sometimes slight, at others very severe. I could not account for it. There was no smell in the lochia indicating the remains of the membranes, or a clot. Nor could I detect anything of the kind by the examination per vaginam. Five grains camphor, and one-eighth morphine were the only means that gave relief. Larger doses of morphine narcotized her too much.

5. Mrs. G—— was confined Dec. 2d, 1852. First labor; did not last an hour. Being absent, another physician was called; birth before he arrived; he removed the after-birth. Calling next day, I found her suffering much from after pains, and some excitement. Left portions of morphine to be used as occasion demanded.

*4th.* Found her worse; high fever; pulse 130; skin hot; abdomen swelled and painful; womb hard; right groin giving much pain on pressure; bowels confined. Directed senna to be used until free purgation; then to use oil of turpentine, 25 drops, and sulphate of morphia one-fourth grain every three hours.

*5th.* Found her much easier, less pain and swelling; pulse still frequent; continue turpen. and morph., with fomentations. Blue mass at night, x. grs. *6th.* Still some fever and pain; pulse 120; tongue coated; no operation; abdomen still enlarged, but not so tender. Omitted turpentine and morphia. Directed a purgative, and spirits nitre, and wine ipecac. every four hours; calomel, ipecac. and camphor pill at night. This case continued to alternate for some two weeks. The lochia continued, sometimes almost ceasing, and then too free. Secretion of milk ceased after the fifth day. The oil of turpentine was not used after the sixth day, excepting when there was much increase of the pain of the womb, when a few doses almost invariably gave relief. The general treatment after this day was fomentations, warm hip bath, spirits nitre and ipecac., when there was fever. Laxatives.

*Dec.* 20. She was entirely convalescent.

6. Mrs. A——, confined February, 10, 1853. Tedious labor. Had pain in the side during labor. Twelfth morning was suddenly taken with severe pain in back; no fever. Camphor and turpentine on cotton applied to it, relieved the pain in the course of an hour. At noon the womb became very painful; skin hot; pulse 110; headache. Directed to take 30 drops spirits turpentine every two hours until relieved. She took three portions with entire relief of the womb.

*Remarks.*—The ages of these cases varied from eighteen to thirty-two. It will be seen that, in all of them but one, the turpentine was commenced early in the attack. The negro woman had been sick several days before she was seen. I am satisfied that this treatment will be successful as it is promptly used. If the remedy is delayed until the inflammation has advanced to structural lesion, it will have to be withdrawn, or, at any rate, combined with mercurials and alteratives with opium.

